# TCF7L2 Polymorphism rs7903146 Is Associated with Coronary Artery Disease Severity and Mortality

**DOI:** 10.1371/journal.pone.0007697

**Published:** 2009-11-17

**Authors:** André Gustavo P. Sousa, Guilherme F. Marquezine, Pedro A. Lemos, Eulogio Martinez, Neuza Lopes, Whady A. Hueb, José E. Krieger, Alexandre C. Pereira

**Affiliations:** 1 Laboratory of Genetics and Molecular Cardiology, Heart Institute (InCor), University of São Paulo Medical School, São Paulo, Brazil; 2 Clinical Medicine Department, Federal University of Rio Grande do Norte, Natal, Brazil; 3 Hemodynamic Laboratory, Heart Institute (InCor), University of São Paulo Medical School, São Paulo, Brazil; 4 Coronary Artery Disease Service, Heart Institute (InCor), University of São Paulo Medical School, São Paulo, Brazil; National Institute of Child Health and Human Development/National Institutes of Health, United States of America

## Abstract

**Background:**

TCF7L2 polymorphisms have been consistently associated with type 2 diabetes mellitus in different populations and type 2 diabetes mellitus is a major risk factor for cardiovascular disease, especially coronary artery disease. This study aimed to evaluate the association between TCF7L2 polymorphism rs7903146 and coronary artery disease in diabetic and non-diabetic subjects.

**Methods and Results:**

two populations were studied in order to assess severity of coronary artery disease and cardiovascular events incidence. Eight-hundred and eighty nine subjects who were referred for cardiac catheterization for coronary artery disease diagnosis were cross-sectionally evaluated for coronary lesions (atherosclerotic burden) and 559 subjects from the MASS-II Trial were prospectively followed-up for 5 years and assessed for major cardiovascular events incidence. As expected, rs7903146 T allele was associated with diabetes. Although diabetic patients had a higher prevalence of coronary lesions, no association between TCF7L2 genotype and coronary lesions was found in this subgroup. However, non-diabetic individuals carrying the T allele were associated with a significantly higher frequency of coronary lesions than non-diabetic non-carriers of the risk allele (adjusted OR  = 2.32 95%CI 1.27–4.24, p = 0.006). Moreover, presence of multi-vessel coronary artery disease was also associated with the CT or TT genotypes in non-diabetics. Similarly, from the prospective sample analysis, non-diabetics carrying the CT/TT genotypes had significantly more composite cardiovascular end-points events than CC carriers (p = 0.049), mainly due to an increased incidence of death (p = 0.004).

**Conclusions:**

rs7903146 T allele is associated with diabetes and, in non-diabetic individuals, with a higher prevalence and severity of coronary artery disease and cardiovascular events. name of registry site (see list below), registration number, trial registration URL in brackets.

**Clinical Trial Registration Information:**

Medicine, Angioplasty, or Surgery Study (MASS II): Unique identifier: ISRCTN66068876.

## Introduction

Diabetes mellitus (DM) is a common disease present in about 200 million of individuals worldwide and its prevalence has been increasing [1]. People with DM have a higher risk of cardiovascular (CV) complications, including coronary artery disease (CAD). Actually, diabetes confers an equivalent risk to previous myocardial infarction of future cardiovascular disease (CVD) [2].

Genome-wide association studies have disclosed chromosomic regions containing DM susceptibility genes in chromosome 10q, which were later ascribed to intronic variations in the transcription factor 7-like 2 (TCF7L2) gene and TCF7L2 variants have been consistently associated with type 2 diabetes (T2D) in different populations [3], including the Brazilian population [4]. In addition, associations between TCF7L2 polymorphisms and other conditions such as prostate, breast and colon cancers [5], [6], [7] and even cardiovascular events [8] have been studied.

Thus, we aimed to study the association between the rs7903146 polymorphism, the most widely studied TCF7L2 genetic marker, and CVD in individuals with and without diabetes. For this, we have used two independent populations: 1) individuals referred to cardiac catheterization for suspected coronary artery disease; 2) individuals with established multivessel coronary artery disease from the MASS-II Trial (ISRCTN66068876), whom were followed-up for 5 years for CV events incidence (all cause mortality, myocardial infarction, recurrent angina, and new cardiac procedures).

## Methods

### Patient populations

#### Individuals referred for cardiac catheterization for the diagnosis of CAD

A cross-sectional study of severity of coronary artery disease was performed at the Laboratory of Hemodynamic of the Heart Institute (InCor), São Paulo, Brazil. All patients had a suspected clinical diagnosis of angina pectoris and stable angina and no patient enrolled in this study was currently experiencing an acute coronary syndrome. Patients with previous cardiac events and/or previous diagnostic or therapeutic coronary catheterization, acute ischemic events, heart failure class III–IV, hepatic dysfunction, familiar hypercholesterolemia, previous heart or kidney transplantation, and in anti-retroviral treatment were excluded from the study. Of a total of 1,451 patients consecutively seen, 167 were excluded because they have been previously submitted to cardiac bypass surgery, 140 were excluded because they have already received coronary stents, 78 have been previously submitted to angioplasty, 163 due to a diagnostic cardiac catheterism before this evaluation and 7 because of genotyping missing data. The coronary lesions were evaluated by an expert team by analyzing angiogram images, based on standard protocols. Patients answered a clinical questionnaire that covered questions regarding their personal medical history, family history of CAD, sedentary behavior, smoking status, hypertension, obesity, dyslipidemia, diabetes, and current medical treatment. During the interview, anthropometric measures were collected (weight and height), and a blood sample during cardiac catheterization for genomic DNA extraction was obtained. All patients signed an informed consent form and the study has been approved by the Ethics Committee of the Heart Institute of the University of Sao Paulo-Brazil (research protocol # 1081/07).

#### MASS II Study population

The participants of this second population were selected from the prospective, randomized, controlled clinical trial MASS II. The MASS II trial was designed to compare medical treatment, angioplasty/stent placement, and surgical myocardial revascularization in patients with stable multivessel CAD with preserved left ventricular function. Briefly, 2,076 candidates who were indicated for myocardial revascularization were evaluated from May, 1995 to May, 2000. Of these, 611 patients were eligible and met all entry criteria to be randomly assigned to one of the three therapeutic groups. The inclusion criteria were symptomatic multivessel coronary disease, preserved left ventricular function, and the presence of coronary lesions (70% of stenosis) amenable to angioplasty. Then patients were followed-up for five years and were assessed for cardiovascular events such as mortality, myocardial infarction, angioplasty, myocardial revascularization surgery and the combination of them. All patients from the MASS-II study were repeatedly evaluated in a single-center (Heart Institute, Brazil) by the same medical team for the five years of follow-up, according to a standard protocol. All events (except non-hospital deaths) were preferentially attended at the Heart Institute [9], [10], [11].

All patients signed an informed consent form and the study has been approved by the Ethics Committee of the Heart Institute of the University of Sao Paulo-Brazil (research protocol # 1081/07).

### Coronary artery disease severity score

A score was created in order to assess the severity of the CAD. Twenty coronary segments were analyzed for the construction of the score. Each epicardial vessel or main branch was divided into 3 segments (proximal, medial and distal) except secondary branches of the right coronary artery that were divided in 2 segments, proximal and distal. The score was defined as the sum of each coronary segment analyzed: no lesion or irregularities — 0 points, less than 50% lesion — 0.3 points, 50–70% lesion — 0.6 points, 70–90% lesion — 0.8 points and 90–100% lesion — 0.95 points. Use and characteristics of this score has been previously described [12].

### TCF7L2 rs7903146 polymorphism genotyping

Genomic DNA was extracted from peripheral blood leukocytes by means of a standard salting-out procedure. PCR primers used were: 5′ ACA ATT AGA GAG CTA AGC ACT TTT TAG GTA 3′ and 5′ GTG AAG TGC CCA AGC TTC TC 3′. The studied polymorphism was detected by polymerase chain reaction-restriction fragment length polymorphism assay (PCR-RFLP). Briefly, a 30-cycle PCR was carried out in a PTC-DNA Engine Tetrad2 using a 10 µL reactive solution containing 10 mM Tris-HCl (pH 9.0), 50 mM KCl, 2.5 mM MgCl2, 100 µM of each dNTP, 0.3 U Easy Taq DNA Polymerase, 5 pmol of each primer and 1 µL of genomic DNA template. PCR products were digested with 1 U of *Rsa*I restriction enzyme and visualized by 3% agarose gel electrophoresis. The quality control for these assays was assessed by randomly selecting 40 samples that were re-genotyped by an independent technician. Observed concordance between genotyping assays was 100%.

### Statistical analysis

Hardy–Weinberg equilibrium for the distribution of genotypes was estimated by the Chi-square test. Chi-square tests, t-tests, and analysis of variance were used for baseline comparisons. The odds ratios for different association models were calculated with 95% confidence interval (CI) by multiple logistic regression with confounders determined by a backward conditional elimination method for a significance level below 0.05. Long-term survival comparisons were conducted for the entire study group and for the subgroups of diabetic and non-diabetic patients and TCF7L2 genotype. Logistic regression was used to estimate the cross-sectional association of these variables with each of the MASSII trial endpoints (death, myocardial infarction, and recurrent ischemia requiring revascularization), as well as the combined endpoint after the 5-year follow-up period. Survival curves were calculated with the Kaplan-Meier method, and differences between the curves were evaluated with the log-rank statistic. We assessed the relationship between baseline variables and composite end points by using a Cox proportional hazards survival model. A value of p<0.05 was considered significant for comparisons. SPSS version 13 program for Windows was used for statistical analysis.

The authors had full access to the data and take responsibility for its integrity. All authors have read and agree to the manuscript as written.

## Results

### Cross-sectional analysis - Assessment of CAD Severity

In this first part, we studied a population referred for cardiac catheterization for the diagnosis of CAD at the Laboratory of Hemodynamics, InCor, Brazil. Eight hundred and ninety six consecutive patients submitted for the first time only to coronary angiography to study a clinical suggestive diagnosis of coronary artery disease were selected. This study population had 52.4% of male individuals, with a mean age of 59.5 years. The prevalence of hypertension, diabetes, obesity (BMI >30.0 kg/m^2^), smoking, heart failure (HF) class I-II, peripheral artery disease (PAD) and chronic renal failure (CRF) was 73%, 32.1%, 28%, 37.1%, 1.8%, 9.2% and 2.7%, respectively. The mean values of fasting glycemia, total cholesterol, LDL-cholesterol, HDL-cholesterol and triglycerides measurements were 130.2 mg/dl, 229.0 mg/dl, 145.0 mg/dl, 44.8 mg/dl and 181.4 mg/dl, respectively.

In the entire population, the rs7903146 genotype frequencies were: CC  = 46.8% (n = 419); CT  = 42.6% (n = 382); TT  = 10.6% (n = 95). For the CC, CT and TT genotypes, respectively, the prevalence of diabetes was 32.2%, 30.9% and 36.8%. There was no difference in genotype frequencies for age, sex, smoking, BMI, total cholesterol, HDL- and LDL-cholesterol, triglycerides or systolic and diastolic blood pressure, hypertension, APD, HF and CRF.

Although clinically suspected, 267 patients had no identifiable coronary lesions by angiography. As expected, diabetic patients had a higher prevalence of obstructive coronary lesions (∼80% of individuals), independent of the genotype they carry (p = 0.93). Although there were no differences in the prevalence of coronary lesions between the three TCF7L2 genotypes in the entire population, non-diabetic patients that carried allele T (non-diabetics CT and TT individuals) had a significantly higher frequency of coronary lesions as compared to non-diabetic individuals with the CC genotype. Allelic analysis also revealed a significant association between allele T and coronary lesions in this subgroup (OR  = 1.37 per allele, 95%CI  = 1.05–1.78, p = 0.025). The demographic and laboratorial characteristics of non-carries of T allele and carries are shown in Table 1. The individuals CT/TT had an almost 2.5 fold higher chance of having obstructive coronary lesions when compared to group 1 (Table 2). This increased odd of having an increased atherosclerotic burden was still present even after adjustment for age, sex, BMI, smoking, hypertension, glycemia, total cholesterol, LDL-c, HDL-c and triglycerides and use of statins, aspirin and beta-blockers.

**Table 1:** Demographic and laboratorial characteristics of non-diabetic and diabetic subjects from the cross-sectional population according to TCF7L2 genotype.

	Non-diabetic subjects	Diabetic Subjects
	CC (n = 284)	CT + TT (n = 324)	P value*	CC (n = 135)	CT+TT (n = 153)	P value*
Age (years)	58.4±11.1	59.4±10.4	0.229	61.0±9.6	60.3±9.4	0.507
Male gender (%)	53.5	57.1	0.376	45.9	46.4	0.935
Smoking (%)	35.6	41.7	0.123	29.6	36.6	0.210
Sedentarism (%)	94.0	90.1	0.214	94.8	93.5	0.303
BMI (kg/m^2^)	27.8±5.3	27.3±4.6	0.087	28.9±5.5	28.6±5.4	0.672
Hypertension (%)	70.1	65.7	0.254	80.7	86.3	0.205
Congestive Heart Failure (%)	10.2	8.6	0.508	6.7	15.7	0.016
Chronic Kidney Disease (%)	1.8	1.5	0.770	5.2	3.9	0.755
Glucose (mg/dL)	104.2±21.1	103.9±19.2	0.288	172.1±69.1	177.7±73.2	0.522
Total cholesterol (mg/dL)	229.1±47.3	230.3±53.3	0.510	231.2±47.4	223.9±47.0	0.248
LDL-c (mg/dL)	145.4±38.5	147.1±43.1	0.698	154.0±42.3	132.4±41.2	0.002
HDL-c (mg/dL)	46.0±13.4	44.7±12.9	0.577	42.9±10.4	44.6±13.0	0.394
Triglycerides (mg/dL)	164.7±104.5	163.3±102.5	0.831	223.4±192.5	210.2±188.1	0.602

(*) Comparison between genotypes groups in non-diabetic and diabetic subjects, calculated by chi-square test for categorical variables and by t-student test for continuous variables; BMI - body mass index.

**Table 2:** Prevalence and risk estimative of coronary lesions according to TCF7L2 genotype.

	Genotypic groups	Prevalence (n)	OR	Adjusted OR*	P value
*Diabetic subjects*	CC	80.7% (109)	1.00	-	0.727
	CT+TT	79.1% (121)	0.902 (0.51–1.61)	-	
*Non-diabetic subjects*	CC	60.6% (172)	1.00	1.00	0.006†
	CT+TT	70.1% (227)	1.52 (1.09–2.13)	2.32 (1.27–4.24)	
*All subjects*	CC	67.1% (281)	1.00	1.00	0.112†
	CT+TT	73.0% (348)	1.325 (0.99–1.76)	1.47 (0.91–2.36)	

(*) Adjusted for sex, BMI, smoking, hypertension, glycemia, total cholesterol, LDL-c, HDL-c, triglycerides.

(†) p value for adjusted OR.

Regarding atherosclerosis burden, non-diabetic carriers of the T allele had a significantly higher chance of having any coronary lesion (OR 1.51, p = 0.01); a significantly higher prevalence of three-vessel coronary disease as opposed to having normal coronary arteries (OR 1.76, p = 0.01) (Table 3). In addition, considering the severity score divided in tertiles, having the CT or TT genotypes was also associated with an increased odd of being in the second or third tertile of atherosclerosis burden (Table 4). By linear regression, the presence of one T allele increased the score value (analyzed here as a continuous variable) in 1.4 points (p = 0.038). After adjustment for potential confounders, including glycemia, some, but not all, unadjusted comparisons were no longer statistically significant (Tables 3 and 4).

**Table 3:** Distribution of coronary lesions in non-diabetic patients with coronary lesions according to genotypes.

	CC % (n)	CT + TT % (n)	OR*	P value	Adjusted OR†	P value‡
Normal	43.7 (124)	34.0 (110)	1.00		1.00	
One-vessel disease	20.8 (59)	27.8 (90)	1.72 (1.13–2.61)	0.01	2.09 (1.08–4.04)	0.03
Two-vessel disease	19.4 (55)	16.0 (52)	1.07 (0.67–1.68)	0.79	1.47 (0.75–2.90)	0.26
Three-vessel disease	16.2 (46)	22.2 (72)	1.76 (1.12–2.77)	0.01	2.06 (1.00–4.25)	0.05

(*) Odds Ratio calculated by comparison between one-vessel, two-vessel, and three-vessel disease versus normal coronary arteries by multiple logistic regression.

(†) Adjusted for sex, BMI, smoking, hypertension, glycemia, total cholesterol, LDL-c, HDL-c, triglycerides and use of statins, aspirin and beta-blockers; (‡) p value for adjusted OR.

**Table 4:** Tertile distribution of severity artery coronary lesions score (SCS) in non-diabetic patients according to TCF7L2 genotypes.

	CC % (n)	CT + TT % (n)	OR*	P value	Adjusted OR†	P value‡
1st Tertile (≤0.9)	38.7 (110)	28.4 (92)	1.00		1.00	
2^nd^ Tertile (0.91–2.2)	29.9 (85)	36.1 (117)	1.65 (1.11–2.44)	0.01	1.004 (0.53–1.91)	0.99
3^rd^ Tertile (≥2.2)	31.3 (89)	35.5 (115)	1.55 (1.04–2.28)	0.03	1.31 (0.69–2.47)	0.40

(*) Odds Ratio calculated by comparison between tertiles by multiple logistic regression (reference group first tertile).

(†) Adjusted for sex, BMI, smoking, hypertension, glycemia, total cholesterol, LDL-c, HDL-c, triglycerides, use of statins, aspirin and beta-blockers; (‡) p value for adjusted OR.

### Prospective Analysis - Incidence of Cardiovascular Events in the MASS-II Trial

In order to evaluate if TCF7L2 genotype was also associated with cardiovascular events in a population of individuals already with extensive coronary artery disease, we studied the patients from the MASS-II Trial. This study population included 559 eligible individuals, of whom 69.2% were male, with a similar mean population age of 59.7 years and mean BMI of 27.1 kg/m^2^. Hypertension was present in 59.6%, diabetes in 30.9% and smoking in 33.9% of studied individuals. The mean value of fasting glycemia, total cholesterol, LDL-cholesterol, HDL-cholesterol and triglycerides measurements were 129.6 mg/dl, 223.4 mg/dl, 147.5 mg/dl, 37.3 mg/dl and 194.0 mg/dl, respectively. Genotype counts and frequency were as follow: CC  = 159 (28.4%), CT  = 336 (60%), and TT  = 65 (11.6%). The characteristics of carriers of T allele and non-carriers patients are shown in Table 5.

**Table 5:** Demographic and laboratorial characteristics of non-diabetic subjects from the MASS-II population according to TCF7L2 genotype.

	Non-diabetic subjects	Diabetic subjects
	CC (n = 120)	CT + TT (n = 266)	P value*	CC (n = 38)	CT + TT (n = 135)	P value*
Age (years)	59.6±9.2	59.1±9.4	0.836	62.7±6.4	59.8±9.1	0.071
Male gender (%)	68.3	71.4	0.537	60.5	65.2	0.280
Smoking (%)	34.2	36.5	0.663	31.6	25.9	0.479
BMI (kg/m^2^)	27.6±3.9	26.4±4.0	0.966	27.9±4.4	28.0±4.6	0.880
Hypertension (%)	56.7	53.8	0.595	73.7	66.7	0.673
Previous MI (%)	39.2	50.0	0.048†	26.3	45.9	0.030
Glucose (mg/dL)	104.0±17.4	107.2±21.3	0.551	161.3±63.6	183.0±79.7	
Total cholesterol (mg/dL)	220.5±44.4	225.9±48.3	0.409	221.0±49.6	219.2±50.6	0.848
LDL cholesterol (mg/dL)	147.3±38.2	149.9±44.7	0.349	144.7±41.9	141.2±46.8	0.688
HDL cholesterol (mg/dL)	37.5±9.4	37.0±10.7	0.783	37.2±11.9	38.1±10.6	0.673
Triglycerides (mg/dL)	186.2±109.9	185.0±94.6	0.191	213.3±156.2	205.4±141.5	0.765

(*) calculated by qui-square test for categorical variables and by t-student test for continuous †variables; (†) p value <0.05; BMI - body mass index; MI - myocardial infarction.

**Figure 1:**
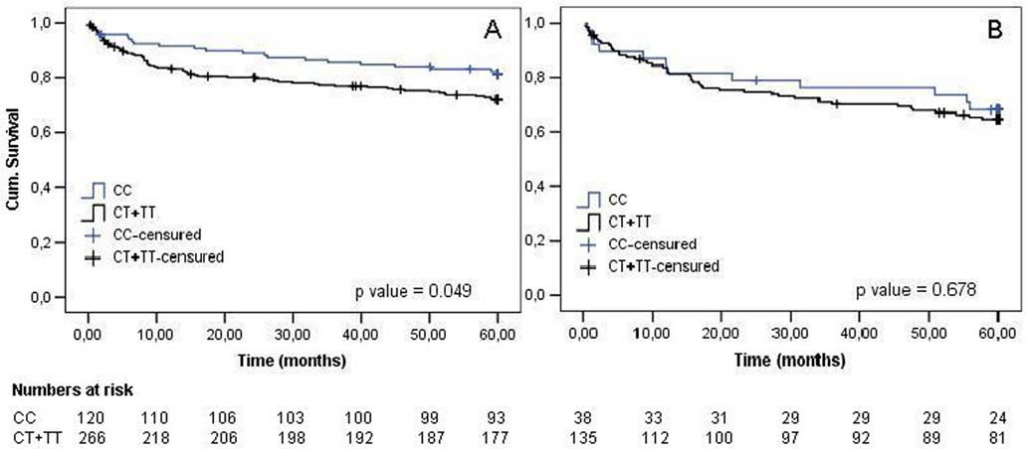
Kaplan-Meier Curve of TCF7L2 genotypes and composite cardiovascular end points in non-diabetic (A) and diabetic patients (B) after 5 years of follow-up. In non-diabetic patients, individuals CT and TT had a significant higher incidence of composite cardiovascular endopoints (death, non-fatal myocardial infarction, refractory angina requiring revascularization or new cardiac catheterization – PCI). Although diabetic individuals had a higher incidence of cardiovascular events than non-diabetics, a presence of T allele was not associated with cardiovascular endpoints.

Kaplan-Meier curve analysis is shown in Figure 1. The group with rs7903146 genotype CT or TT was associated with a significantly higher incidence of cardiovascular events (Figure 1A). In absolute values, CT/TT group had 47 composite events, compared to 12 events in the CC group. However, it can be observed that the TCF7L2 polymorphism is not associated with the composite end point of cardiovascular events (cardiac death, myocardial infarction, and refractory angina requiring revascularization or new cardiac catheterization) in diabetic subjects (Figure 1B). Decompounding the end points in non-diabetic individuals, we observed that the association between TCF7L2 genotype and cardiovascular events occurred mainly because an increased all-cause mortality incidence in individuals harboring the T allele (number of deaths: CT/TT  = 27 *vs* CC  = 4, p value  = 0.004). Although incidence of myocardial infarction, myocardial revascularization, percutaneous coronary intervention (PCI) and stroke were higher in CT/TT group in non-diabetics, we were not able to reach statistical significance (p>0.10). Finally, after adjustment for several established risk factors for cardiovascular disease listed in Table 5, only TCF7L2 genotypes CT or TT (p = 0.047), age (p = 0.003) and smoking (p = 0.013) were significantly and independently associated with cardiovascular events incidence.

## Discussion

The study data showed a significant association between TCF7L2 rs7903146 genotypes and coronary artery disease in non-diabetic individuals, in both independent populations. Interestingly, we were not able to demonstrate this association in diabetic patients.

It has been well established for many decades that diabetes increases the risk of CVD. About 65% of deaths in individuals with type 2 diabetes are related to heart disease or stroke [13], [14]. These data are concordant with the results of this study, in which diabetic individuals had a higher severity of CAD and cardiovascular events incidence than non-diabetics. Considering this information and since there were no associations between rs7903146 polymorphism and established CV risk factors, other than diabetes itself, we hypothesize that the association between TCF7L2 genotype and cardiovascular phenotypes occurs due to glycemic homeostasis changes. Nevertheless, given the scenario of established diabetes, coronary disease risk will be increased by itself, despite TCF7L2 genotype status.

Genetic variants in the gene encoding for TCF7L2 have been associated with type 2 diabetes and impaired cell function, but the mechanisms still remain unknown. The gene TCF7L2 is located on chromosome 10 and encodes the transcription factor4 (TCF-4) and single nucleotide polymorphism (SNP) rs7903146 strongly predicts prevalent and future T2DM in several independent populations [3]. The risk T allele was associated with impaired insulin secretion and incretin effects [15], trending to postprandial hyperglycemia. Data from MONICA/KORA surveys revealed the T allele at rs7903146 as inversely associated with log-transformed HOMA-%Beta (as a measure of basal insulin secretion), but no association was found with insulin resistance and metabolic syndrome. Therefore, we hypothesize that even non-diabetic carriers of CT/TT genotypes have increased postprandial glycemia and consequently, higher CAD tendency, severity and cardiovascular events incidence.

Actually, a meta-analysis of published data from 20 studies with almost 100,000 subjects followed-up for 12.4 years confirmed a significant association between 2-hour glucose levels after an oral glucose overload and incident cardiovascular events [16]. In this same study, there was a suggestive trend (p value equal to 0.056) between fasting glucose and cardiovascular events. These data support the hypothesis that even non-diabetic degrees of fasting and mainly postprandial hyperglycemia are associated with CVD. Therefore, if T allele is associated with postprandial hyperglycemia, we hypothesized T allele would be associated with cardiovascular events. Plasma glucose is a continuous risk factor for CVD, in both diabetic and non-diabetic people, the risk extending below impaired fasting and impaired glucose tolerance cutoffs. Indeed, some authors consider that dysglycemia should be added to the list of established continuous cardiovascular risk factors [17]. Most cardiovascular risk factors are affected directly by an acute increase of glycemia: increase in LDL oxidation and endothelial dysfunction (vasoconstriction and decreased vasodilating response), increased production of collagen from the mesangial cell, activation of blood coagulation, increase in blood pressure, increase in the circulating levels of intracellular adhesion molecule 1 (ICAM-1), increase in inflammation (production of plasma interleulin-6, interleukin-18 and tumor necrosis factor-α - TNF-α) and increase in oxidative stress [17], [18]. Unfortunately, there was no information about glycated haemoglobin or postprandial glycemia in our study and we were not able to evaluate this correlation. Regarding fasting glycemia, there is no difference between genotypes in non-diabetic individuals, but we were not able to evaluate the effects of the studied genotype in longitudinal glucose-related variables. In addition, after adjustment for other established cardiovascular risk factors, including baseline fasting glycemia, genotypes CT and TT continued to be related to CAD severity and CV events, mainly death.

Moreover, some studies have observed that transcriptional activation of TCF-4 is related to the nuclear factor-κB (NF-κB) signaling pathway, which regulates inflammatory signaling pathways [19] and this could be hypothesized as an additional pleiotropic mechanism operant in the *milieu* of the vascular wall.

It should be remembered that the diabetic subgroups from both populations have a smaller number of individuals and one can not exclude that this small number of individuals associated with the higher prevalence of coronary artery disease and incidence of cardiovascular events in this particular subgroup of individuals has led to reduced statistical power to detect the association we were able to describe on non-diabetic individuals. However, even a relatively small sample size of non-diabetic subjects was able to provide an adequate statistical power analysis, in part due to the relatively large effect size. Despite this, it is not possible to exclude completely the possibility of false positive results. Considering it is a genetic study of association, a limitation is the possibility of other non evaluated variable that, by acting as a confounder, could play a significant role in the findings. One should remember, however, that multiple potential confounder adjustments were made in the presented analysis. Finally, results in the same direction in two different and independent populations may actually be indicative of a true association.

Previous myocardial infarction was significantly more prevalent in genotypes CT and TT in non-diabetic individuals from the MASS-II population. However, this factor was not independently associated with CV events, like TCF7L2 genotype, smoking and aging. Therefore, the higher prevalence of a previous myocardial infarction at baseline does not completely explain such association. Actually, these data contribute to the idea that non-diabetic patients carriers of the rs7903146 allele T presents a higher CAD severity.

Bielinski *et al* [8] have recently published a study in which TCF7L2 SNPs rs7903146, rs12255372, rs7901695, rs11196205 and rs7895340 were assessed with a similar objective, using more than 13,000 subjects from the ARIC study population. In their report, they have not demonstrated a significant association between any TCF7L2 SNP with incident coronary disease, ischemic stroke, CVD, prevalent peripheral artery disease (PAD) or all-cause mortality in the full cohort nor when stratified by race or diabetic status. Although divergent results, there are large methodological differences between the two studies: in the ARIC study, all patients with prevalent coronary disease were excluded from analysis and, consequently, the patients were free of prevalent CVD at baseline. On the other hand, our study populations contain patients with suspected CAD (cross-sectional study population) or with established multi-vessel CAD plus stable angina (MASS-II cohort) at baseline and these differences in the *a priori* risk of coronary artery disease in these populations could explain the apparent conflicting results. Here as well, it should be acknowledged that the interpretations of the present study are derived from two different and independent cohorts of patients, straightening the conclusions of the present work.

In conclusion, these data demonstrate an association between coronary artery disease severity and rs7903146 polymorphism mainly in non-diabetic individuals with increased cardiovascular risk. In people with diabetes, we were not able to show this association. Additionally, it was found that non-diabetic allele T carriers also had higher cardiovascular event incidence, mainly due to death. Due to the limitations already discussed, more studies are necessary to confirm this association and to evaluate the intrinsic mechanisms underlining this process. If this association is confirmed, other studies will also be useful to establish the best strategy to use this information for the better risk stratification of individuals.
